# Nonclonal chromosomal alterations and poor survival in cytopenic patients without hematological malignancies

**DOI:** 10.1186/s13039-019-0458-9

**Published:** 2019-11-12

**Authors:** Osamu Imataki, Hiroyuki Kubo, Akihiro Takeuchi, Makiko Uemura, Norimitsu Kadowaki

**Affiliations:** 1 Division of Hematology, Department of Internal Medicine, Faculty of Medicine, Kagawa University, 1750-1 Ikenobe, Miki-cho, Kita-gun, Kagawa, 761-0793 Japan; 2 Department of Clinical Laboratory, Kagawa University Hospital, Kagawa, Japan

**Keywords:** Clonal chromosomal alterations, Nonclonal chromosomal alterations, Chromosomal instability, Idiopathic cytopenia without undetermined significance

## Abstract

**Background:**

Clonal chromosomal alterations (CCAs) reflect recurrent genetic changes derived from a single evolving clone, whereas nonclonal chromosomal alterations (NCCAs) comprise a single or nonrecurrent chromosomal abnormality. CCAs and NCCAs in hematopoietic cells have been partially investigated in cytopenic patients without hematological malignancies.

**Methods:**

This single-center retrospective study included 253 consecutive patients who underwent bone marrow aspiration to determine the cause of cytopenia between 2012 and 2015. Patients with hematological malignancies were excluded. CCA was defined as a chromosomal aberration detected in more than two cells, and NCCA was defined as a chromosomal aberration detected in a single cell.

**Results:**

The median age of the patients was 66 years. There were 135 patients without hematological malignancies (median age, 64 years; 69 females); of these, 27 patients (median age, 69 years; 8 females) harbored chromosomal abnormalities. CCAs were detected in 14 patients; the most common CCA was −Y in eight patients, followed by inv.(9) in three patients and mar1+, inv. (12), and t (19;21) in one patient each. NCCAs were detected in 13 patients; the most frequent NCCA was +Y in four patients, followed by del (20), + 8, inv. (2), − 8, and add (6) in one patient each. Moreover, nonclonal translocation abnormalities, including t (9;14), t (14;16), and t (13;21), were observed in three patients. One patient had a complex karyotype in a single cell. The remaining 106 patients with normal karyotypes comprised the control group (median age, 65 years; range, 1–92 years; 56 females). Further, follow-up analysis revealed that the overall survival of the NCCA group was worse than that of the CCA and the normal karyotype groups (*P* < 0.0001; log-rank test).

The survival of the NCCA-harboring cytopenic patients was worse than that of the CCA-harboring cytopenic patients without hematological malignancies, suggesting that follow-up should be considered for both CCA- and NCCA-harboring cytopenic patients.

## Introduction

Cytogenetic analysis of karyotype aberrations aims to determine genetic alterations in congenital disorders and acquired oncogenesis in tumor cells. In medical oncology, chromosomal analysis is used for the diagnosis or prediction of prognosis [1]. The characterization of malignancies, such as determining the grade of malignancy, is an alternative use of chromosomal analysis in cancer genetics. Clonal chromosomal alterations (CCAs) are recurrent genetic changes derived from a single evolving clone and conventionally represent the genetic profile of cancer progression [2], which effectively explains the model for solid malignancies [3]. Conversely, nonclonal chromosomal alterations (NCCAs) comprise a single or nonrecurrent chromosomal abnormality. Both CCAs and NCCAs have been detected in several cancers such as hematological malignancies and solid tumors. In the cancer progression model, CCAs signify highly canonical oncogenesis to elucidate cancer pathogenesis [1]. Primarily, CCAs are considered as oncogenic genotypic changes that occur in patients with cancer, whereas NCCAs are considered as a stochastic occurrence and background artifact. However, NCCAs have been redefined as reliable index markers for cancer development, and a novel understanding for chromosomal instability (CIN) has therein been explained [1]. In addition, recent research on genetic clonality and CIN in cancer oncology has proposed NCCAs as an underlying mechanism contributing to the cancer development. Specifically, chromosomal instability in daughter cells during the duplication of cancer cells is suggested to the accumulation of new chromosomal abnormalities. In this scenario, a high frequency of CCAs in cancer cells indicates stability whereas a high frequency of NCCAs signifies instability. Furthermore, genomic instability correlates with an increased frequency of NCCAs but not of CCAs. Thus, although the distinctions between CCAs and NCCAs have been comprehensively investigated in association with CIN, the CCA/NCCA ratio in hematopoietic cells of cytopenic patients without hematological malignancies has remained partially investigated. Thus, this study aimed to investigate chromosomal abnormalities in bone marrow cells to elucidate the frequency and variety of the CCA/NCCA ratio in cytopenic patients without hematological malignancies.

## Materials and methods

### Study population and sample preparation

We retrospectively reviewed 253 consecutive patients who underwent bone marrow aspiration to determine the cause of cytopenia between 2012 and 2015 at our institution. The patient bone marrow cells were counted and resuspended in RPMI medium. In addition, chromosomal analysis by the G-banding method was performed at an outside laboratory (SRL, Tokyo, Japan) using unstimulated short-term (24–48-h) cultures of bone marrow cells with trypsin digestion. The results were centrally reviewed by SRL. Furthermore, we determined the types of chromosomal abnormalities based on the 1995 ISCN criteria [4]: (a) CCAs were defined as the presence of at least two of the same chromosomal abnormality among the 20–40 mitotic cells that were analyzed [2]; and (b) NCCAs were defined as the presence of a chromosomal abnormality in a single cell. The 1995 ISCN criteria were also used to determine the presence of clones with numerical and structural abnormalities. Clone identification was necessary in cases of structural abnormalities or hyperdiploids to detect two abnormal metaphases, and at least three abnormal metaphases were necessary for hypodiploids. For cases with no detectable chromosomal abnormalities, the karyotype was considered normal only after a minimum of 20 metaphases were assessed.

### Definitions

The current study included patients with idiopathic cytopenia of undetermined significance (ICUS). The ICUS diagnosis was based on the consensus criteria [5, 6] and included three or more hematology specialists. In addition, the myelodysplastic syndrome (MDS) diagnosis was eliminated for the ICUS diagnosis [5, 7]. Furthermore, the patients had to fulfill the following criteria to be included in the study: bone marrow blast < 5.0%, single or multi-lineage dysplasia < 10%, and no detection of CCA indicating MDS.

### Ethics approval

The Kagawa University Hospital Institutional Review Board (IRB) approved the study and submission of the results for publication. Informed consent to be included in the study was obtained from all subjects under the IRB protocol. This study was conducted in accordance with the ethical standards of the responsible committee on human experimentation (Kagawa University Hospital IRB) and the Helsinki Declaration (1964, amended most recently in 2008) of the World Medical Association.

## Results

**Fig. 1:**
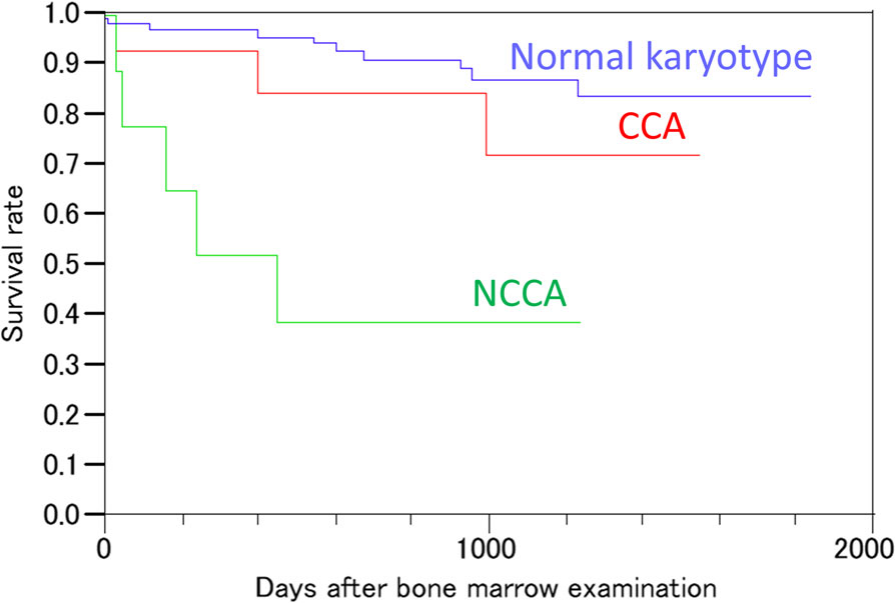
Follow-up of the study patients showing that the overall survival of the NCCA group is worse than those of the CCA and the normal karyotype groups (*P* < 0.0001, log-rank test). CCA, clonal chromosomal alteration; NCCA, nonclonal chromosomal alteration

The current study included 253 bone marrow cases (117 females and 136 males; median age, 66 [range, 1–92] years). There were 135 patients without hematological malignancies (66 males and 69 females; median age, 64 [range, 1–92] years). Of these, 27 patients (20.0%; median age, 69 [range, 54–81] years; 8 females) harbored chromosomal abnormalities. CCAs were detected in 14 patients (10.4%); the most frequent CCA was −Y in eight patients, followed by inv.(9) in three patients and mar1+, inv. (12), and t (19;21) in one patient each. In contrast, NCCAs were detected in 13 patients (9.6%); the most frequent NCCA was +Y in four patients, followed by del (20), + 8, inv. (2), − 8, and add (6) in one patient each. In addition, the nonclonal translocation abnormalities observed in three patients were t (9;14), t (14;16), and t (13;21), which suggested the presence of a malignancy; however, a specific malignancy diagnosis was not reached (Table 1). Of note, only one patient exhibited a complex karyotype in a single cell. The follow-up analysis revealed that the overall survival was worse in the 14 patients with CCAs compared with the 13 patients with NCCAs (Fig. 1). The median follow-up periods were 756 (range, 0–1846), 846 (range, 15–1557), and 91 (0–1246) days in the normal karyotype, CCA, and NCCA groups, respectively. The total of 11 deaths observed in the normal karyotype group included lung cancer, pharyngeal cancer, hepatocellular carcinoma, acute myeloid leukemia, aplastic anemia, polymyositis, Wegener granulomatosis, and myasthenia gravis in 2, 1, 2, 1, 2, 1, 1, and 1 patient, respectively. The causes of the three mortalities in the CCA group were Felty’s syndrome, acute heart failure, and tongue cancer. Finally, the causes of mortality in the NCCA group were polymyositis, lung cancer, and ICUS in 1, 2, and 2 patients, respectively.

**Table 1:** The characteristics of three cases with nonclonal translocation abnormalities

Age	Sex	Specific CCA	Follow-up period (months)	Age of the last follow-up	Outcome	Underlying diseases	Cause of death
76	F	t(13;21)(q11;q11.2)	28	78	Alive	Ureteral tumor, chronic thyroiditis	–
76	M	t(14;16)(p24;q24)	0	76	Alive	Hypertension, diabetes mellitus, angina	–
60	M	t(9;14)(p11.2;q11.2)	4	60	Dead	Acute hepatitis, lymphocytopenia	CMV enteropathy

## Discussion

One recent, previously unappreciated characteristic of NCCA is the clinically meaningful nonrecurrent change in the chromosome [8]. Although NCCAs are not derived from a common single ancestor, they can be a reliable marker for genetic instability. The present study results revealed that chromosomal abnormalities were present in an appreciable subset of cytopenic patients (20.0%, 27/135). While the CCAs were observed in 10.4% (14/135) of the patients, the NCCAs were present in 9.6% (13/135) of the study cohort. In the present consecutive cohort study, the survival rate was lower among the NCCA-harboring cytopenic patients compared with the CCA-harboring cytopenic patients without hematological malignancies. One of the clinical implications of NCCAs in patients with cancer is the associated shorter survival compared with those harboring CAAs [9]. CIN, a critical concept in cancer survival, contributes to genetic variations in cancer and thus may lead to tumor development and drug resistance [9]. In some hematological malignancies, the clinical role of NCCA has been discussed as a CIN-derived prognostic biomarker [10, 11]. As a consequence of cancer chemotherapy, cytotoxic drugs induce damage in both cancerous and normal cells. Therefore, the newly acquired NCCAs that occur after initial remission [10] that are unrelated to pretreatment karyotypes are associated with poor survival [11]. In solid tumor models, intratumor heterogeneity [12] has been recognized as a critical mechanism of evolution through space and time [13]. Until recently, various cancer evolution models have been proposed and, indeed, different cancer evolution models can explain all cancer types including acute myeloid leukemia (AML), MDS, and other solid cancers [14]. However, why cancer evolution is a precedent condition of AML, such as ICUS, remains unclear [14]. Recently, the concept of clonal evolution has been demonstrated in the pathogenesis of AML and MDS [15, 16]. In fact, the disease progression hypothesis is supported by the recent clonal expansion theory in more precise genetic models of MDS pathogenesis [17, 18]. Reportedly, all these theories account for the branched or parallel evolution models introduced in general review articles [14, 19]. However, it remains unclear whether these same clonal evolution models can be adapted to elucidate the transition of MDS to AML. Thus, further studies are warranted for the precise elucidation of this mechanism. The implications of CIN in patients with ICUS are difficult to comprehend because the clinical significance of ICUS is not evident. However, both CCAs and NCCAs might be associated with malignancy as well as cancer morbidity and mortality [11]. Somatic chromosomal mosaicism (SCM), a widely applied concept, explains the genetic diversity in each individual or tumor cell and ensures interindividual/intercellular diversity [20]. A recent review suggested that chromosomal heterogeneity manifesting as SCM was associated with pathophysiology in a variety of human diseases including malignancies, inherited diseases, and healthy conditions [21]. A representative SCM with numerical abnormalities is aneuploidy [22], which is commonly observed in various cancer types [23]. SCM is proposed to arise from somatic cell adaptation to stress [24] and therefore can be considered a biomarker of CIN [22], which mediates cellular evolution leading to clonal evolution. Because the process underlying SCM involves chromosomal abnormality as a molecular mechanism, the theory of oncogenicity can explain the relationship of disease-associated NCCAs with clinical outcomes [22]. Here, SCM/CIN is a combined cytogenetic/molecular mechanism that causes adverse events in the cell, which contributes to clinical significance. Conversely, tissue-specific SCM is another important concept that should be considered. Even though the specific relationship of SCM with ICUS and the underlying mechanism have not been reported [22], the cells of origin in a patient with ICUS theoretically can escape the natural selection pressure and become malignant by acquiring CCAs. Accordingly, we propose the hypothesis that NCCAs in ICUS arise from endogenous cellular selection in response to various stresses, which explains the practical incidence of NCCAs and the current study outcomes. Post-genomic studies for SCM have been dedicated to unveiling the clinical meaning of chromosomal heterogeneity in pathogenetic contexts. CCAs and NCCAs are essential for genomic complexity due to their roles in genome heterogeneity, and their clinical significance is reflected in their involvement in the pathogenesis of a wide spectrum of diseases by mediating interindividual genetic diversity [2, 9, 21]. Conversely, genetic heterogeneity is a necessary component of not only a wide variety of diseases but also development and aging. Indeed, CCAs and NCCAs might be useful indicators of clonal cell evolution. CCAs and NCCAs are essential events for normal cellular function under stress and represent an altered genome, one of which contributes to the early events in carcinogenesis. The current study has several limitations. First, the study cohort included a small rate of survival events. As the number of events causing deaths was low, the mortality and morbidity rates may not be directly associated with cytopenia or associated diseases. Several studies previously evaluated the correlation between CIN and cancer-specific death [25]. However, similar observational studies determining overall survival estimated that death by any cause correlated with the type of the underlying cancer [26]. Thus, the present study results suggest a correlation between poor prognosis and CIN in patients with cytopenia. Second, the current study compared substantially different molecular mechanisms of CCAs and NCCAs. Therefore, the cause of mortality may not be directly associated with survival. As the molecular mechanisms of CCAs and NCCAs are potentially different, their biological contribution to CIN might be different as well [8]. Current evidence suggests that NCCAs play a vital role in increasing the number and variation of cancer cell phenotypes [8]. Moreover, therapy responses might be different among cancer types harboring CCAs and NCCAs, which might be associated with different post-treatment clinical outcomes. Third, the cytogenetic data in the current study have not been supported by molecular cytogenetic techniques such as fluorescence in situ hybridization, array-based comparative genomic hybridization, or single nucleotide polymorphism arrays, which are generally required for studying chromosomes. These molecular cytogenetic studies, which can provide a post-genomic perspective focusing on clinical bioinformatics, should be considered in future studies to complement the current study findings. In conclusion, the role and origin of CCAs and NCCAs are genetic heterogeneity contributing to cell evolution. CCAs and NCCAs can indicate early clinical significance mediated by genetic diversity. The present study findings suggest that CCAs and NCCAs should be considered equally significant in clinical settings. Of note, the practical implications of these two genetic events should be elucidated further based on the observed presence of CIN even in asymptomatic patients.

## Data Availability

The datasets used and analyzed during the current study are available from the corresponding author on reasonable request.

## Abbreviations

CCA: clonal chromosomal alterations
CMV: cytomegalovirus
